# Tic20 forms a channel independent of Tic110 in chloroplasts

**DOI:** 10.1186/1471-2229-11-133

**Published:** 2011-09-30

**Authors:** Erika Kovács-Bogdán, J Philipp Benz, Jürgen Soll, Bettina Bölter

**Affiliations:** 1 Ludwig-Maximilians-Universität München, Department Biologie I, Plant Biochemistry, Grosshaderner Str. 2-4, D-82152 Planegg-Martinsried, Germany; 2 Munich Center for Integrated Protein Science CiPS, Feodor-Lynen-Strasse 25, D-81377 Munich, Germany; 3 Energy Biosciences Institute, University of California Berkeley, Berkeley, CA 94720, USA

## Abstract

**Background:**

The Tic complex (Translocon at the inner envelope membrane of chloroplasts) mediates the translocation of nuclear encoded chloroplast proteins across the inner envelope membrane. Tic110 forms one prominent protein translocation channel. Additionally, Tic20, another subunit of the complex, was proposed to form a protein import channel - either together with or independent of Tic110. However, no experimental evidence for Tic20 channel activity has been provided so far.

**Results:**

We performed a comprehensive biochemical and electrophysiological study to characterize Tic20 in more detail and to gain a deeper insight into its potential role in protein import into chloroplasts. Firstly, we compared transcript and protein levels of Tic20 and Tic110 in both *Pisum sativum *and *Arabidopsis thaliana*. We found the Tic20 protein to be generally less abundant, which was particularly pronounced in Arabidopsis. Secondly, we demonstrated that Tic20 forms a complex larger than 700 kilodalton in the inner envelope membrane, which is clearly separate from Tic110, migrating as a dimer at about 250 kilodalton. Thirdly, we defined the topology of Tic20 in the inner envelope, and found its N- and C-termini to be oriented towards the stromal side. Finally, we successfully reconstituted overexpressed and purified full-length Tic20 into liposomes. Using these Tic20-proteoliposomes, we could demonstrate for the first time that Tic20 can independently form a cation selective channel *in vitro*.

**Conclusions:**

The presented data provide first biochemical evidence to the notion that Tic20 can act as a channel protein within the chloroplast import translocon complex. However, the very low abundance of Tic20 in the inner envelope membranes indicates that it cannot form a major protein translocation channel. Furthermore, the independent complex formation of Tic20 and Tic110 argues against a joint channel formation. Thus, based on the observed channel activity of Tic20 in proteoliposomes, we speculate that the chloroplast inner envelope contains multiple (at least two) translocation channels: Tic110 as the general translocation pore, whereas Tic20 could be responsible for translocation of a special subset of proteins.

## Background

Plastids originate from a single endosymbiontic event involving a cyanobacterium-related organism [1,2]. In the course of endosymbiosis a massive gene transfer occurred, during which most plastidic genes were transferred to the host cell nucleus. Consequently, today the majority of plastidic proteins must be post-translationally imported back into the organelle. So far, two protein translocation complexes have been characterized in the outer and inner envelope (IE) membrane: Toc and Tic (Translocon at the outer/inner envelope membrane of chloroplasts) [3,4]. After passing the outer membrane via the Toc translocon, the Tic complex catalyses import across the IE membrane. So far, seven components have been unambiguously described as Tic subunits: Tic110, Tic62, Tic55, Tic40, Tic32, Tic22 and Tic20 (for a detailed review see [5,6] and references therein).

Tic110 is the largest, most abundant [7-9] and best studied Tic component. It contains two hydrophobic transmembrane-helices at its N-terminus, anchoring the protein in the membrane [8,10], and four amphipathic α-helices in the large C-terminal domain that are responsible for channel formation [11,12]. At the intermembrane space side, Tic110 contacts the Toc machinery and recognizes preproteins [8,13,14]. Moreover, loops facing the stroma provide a transit peptide docking site and recruit chaperones such as Cpn60, Hsp93 and Hsp70 [13-17].

Tic110 is expressed in flowers, leaves, stems and root tissues, indicating a role in import in all types of plastids [14,18]. It is essential for chloroplast biogenesis and embryo development [14]. Heterozygous knockout plants are clearly affected: they have a pale green phenotype, exhibit defects in plant growth, display strongly reduced amounts of thylakoid membranes and starch granules in chloroplasts, coupled with impaired protein translocation across the IE membrane.

Tic20 is a second candidate within the Tic complex that was proposed to constitute a protein translocation channel [19-22]. For instance, Tic20 was detected in a cross-link with the Toc complex after *in vitro *import experiments in pea [21]. In a more recent study, Tic20 was found to form a complex of one megadalton containing a preprotein *en route *into the plastid after mild solubilization of pea and *Arabidopsis *chloroplasts [20], also suggesting its involvement in protein import.

Tic20 is predicted to have four α-helical transmembrane domains, and is thus structurally related to mitochondrial inner membrane translocon proteins, namely Tim17 and Tim23 (TMHMM Server [23] and [21]). Distant sequence similarity was also reported between Tic20 and two prokaryotic branched-chain amino acid transporters [24]. Computational predictions place the N- and C-termini in the stroma (TMHMM Server [23] and [25]), however, there is no experimental evidence for the proposed topology in higher plants. The only indication for a N_in_-C_in _topology is a result of a C-terminal GFP-fusion to a highly divergent member of the Tic20 protein family from *Toxoplasma gondii *[22]. In the same study, *tgtic20 *mutants were analysed for protein import into apicoplasts, a plastid type originating from secondary endosymbiosis, and it was found that also this distant homolog of Tic20 is important, albeit probably as an accessory component.

The *Arabidopsis thaliana *genome encodes four Tic20 homologs: *AtTic20-I*, *-II*, *-IV *and *-V. AtTic20-I *shows the closest homology to *Pisum sativum Tic20 (PsTic20)*. It is present in all plant tissues, and its expression is highest during rapid leaf growth [19]. *AtTic20-I *antisense plants exhibit a severe pale phenotype, growth defects and deficiency in plastid function, such as smaller plastids, reduced thylakoids, decreased content of plastidic proteins, and altered import rates of preproteins [19,26]. Knockouts of *AtTic20-I *are albino even in the youngest parts of the seedlings [27]. The presence of another closely related *Tic20 *homolog (*AtTic20-IV*) may prevent *attic20-I *plants from lethality, since *Tic20-IV *is upregulated in the mutants [26,27]. However, additional overexpression of *AtTic20-IV *can only compensate the observed defects to a very low extent indicating that AtTic20-IV cannot fully substitute for the function of AtTic20-I [26]. Two more distantly related homologs are also present in *Arabidopsis *(*AtTic20-II *and *AtTic20-V*). However, their closest orthologs are cyanobacterial proteins [11], and even though a chloroplast transit peptide is weakly predicted [28], their localization (and function) in the cell remain unknown [29].

Based on structural similarity to channel-forming proteins, cross-links to imported preprotein and protein import defects detectable in the knockdown mutants, it was hypothesized that Tic20 forms a protein translocation channel in the IE membrane [21,24]. Furthermore, a cross-link of a minor fraction of Tic110 to Tic20 in a Toc-Tic supercomplex [19] indicates an association of the two proteins. Therefore, it was proposed that the two proteins possibly cooperate in channel formation. However, there was no cross-link detected between the two proteins in the absence of the Toc complex, making a direct or permanent interaction unlikely [21]. Recently, Tic20 was demonstrated to be a component of a one megadalton translocation complex detected on BN-PAGE after *in vitro *import into pea and *Arabidopsis *chloroplasts [20]. Tic110 could not be observed in this translocation complex, it formed a different, several hundred kilodalton smaller complex, supporting the idea that the two proteins do not associate. However, the expected channel activity of Tic20 has not been demonstrated experimentally yet.

In this work we explored the role of Tic20 in relation to Tic110 in more detail. We analysed the expression of Tic20 in *Pisum sativum *(*PsTic20*) and *Arabidopsis thaliana *(focusing on *AtTic20-I *and *AtTic20-IV*) by quantitative RT-PCR, and compared it directly with the expression of *Tic110 *in both organisms. Furthermore, semi-quantitative immunoblot analyses revealed the absolute amounts of Tic20 and Tic110 in chloroplast envelopes. Moreover, we showed that Tic20 and Tic110 are not part of a mutual complex in isolated pea IE. After the successful expression and purification of Tic20 we were able to experimentally verify its predicted α-helical structure and N_in_-C_in _topology. Finally, we report for the first time that Tic20 forms a cation selective channel when reconstituted into liposomes.

## Results and Discussion

### Tic20 and Tic110 display a differential expression pattern

Due to errors in the annotation of *AtTic20-I*, currently available Affymetrix micro-arrays do not contain specific oligonucleotides for this isoform and therefore cannot be used to investigate the expression levels of *AtTic20-I *[27]. We designed specific primers for *Tic20 *and *Tic110 *in pea and *Arabidopsis *and performed a quantitative RT-PCR (qRT-PCR) analysis to obtain comprehensive and more reliable quantitative data about the expression of *Tic20 *than those available from semi-quantitative analysis and the Massively Parallel Signature Sequencing database [19,26,27].

**Figure 1:**
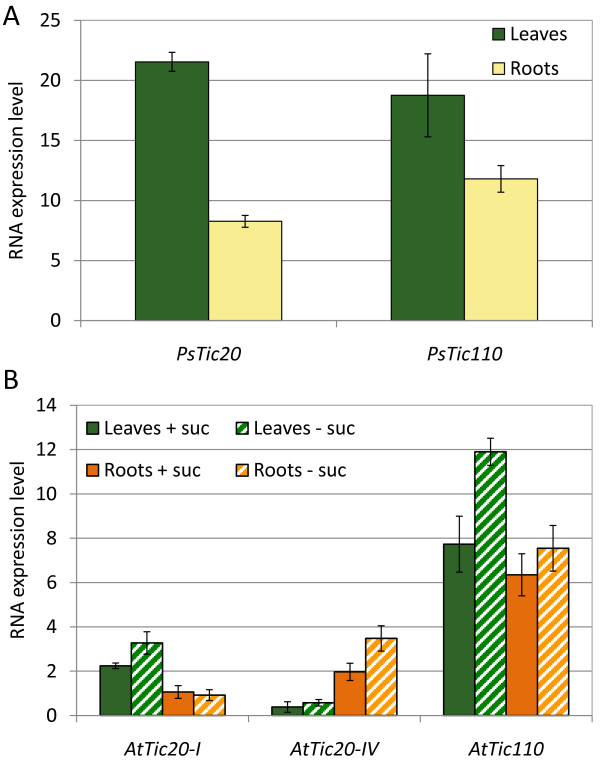
**RNA expression levels of Tic20 and Tic110**. RNA expression levels of (A) *PsTic20*, *PsTic110 *and (B) *AtTic20-I*, *AtTic20-IV *and *AtTic110 *in leaves and roots of two-week-old *Pisum sativum *(Ps) and four-week-old *Arabidopsis thaliana *(At) plants as determined by quantitative RT-PCR using gene-specific primers. Pea plants were grown on soil and *Arabidopsis *plants were cultured hydroponically, the latter in the presence and absence of 1% sucrose (+/- suc). Presented data are the average of at least three measurements.

For the analysis, RNA was isolated from leaves and roots of two-week-old pea seedlings as well as four-week-old *Arabidopsis *plants. *Arabidopsis *was grown hydroponically to provide easy access to root tissue. In all samples, expression of *Tic20 *was analysed in direct comparison to *Tic110 *(Figure 1).

In pea, expression of both genes was found to be lower in root tissue as compared to leaves. In roots, *PsTic110 *RNA is 40% more abundant, while in leaves the expression levels of *PsTic20 *and *PsTic110 *seem to be in a similar range. In *Arabidopsis*, *AtTic20-I *and *AtTic110 *are expressed to a lower extent in roots than in leaves, similar to pea (Figure 1B). These results seemingly contradict those of Hirabayashi *et al. *[26], who concluded a comparable expression level of Tic20-I in shoots and roots. However, they used a non-quantifiable approach in contrast to our quantitative analysis. Furthermore, in our experiments the overall expression of *AtTic20-I *and *AtTic110 *differs notably from that in pea, *AtTic110 *RNA being about 3.5 and 6 times more abundant than *AtTic20-I *in leaves and roots, respectively.

We also designed specific primers for the second Tic20 homolog in *Arabidopsis*, *AtTic20-IV*, and our quantitative method was sufficiently sensitive to precisely define its RNA levels in *Arabidopsis *leaves and roots, allowing direct comparison with the expression of *AtTic20-I *and *AtTic110 *(Figure 1B). Transcription of *AtTic20-IV *had also been investigated in parallel to *AtTic110 *by Teng *et al. *[27], who observed a differential ratio of expression using two different methods, of which one was not even sensitive enough to detect *AtTic20-IV*. A very recent study [26] also investigated the expression of *AtTic20-IV*, however, without any quantification of their data.

Our data show that *AtTic20-IV *is present in leaves and roots with transcript levels similar to *AtTic20-I*, but less abundant than *AtTic110*. Interestingly, and in accordance with the data presented by Hirabayashi *et al. *[26], transcript levels of *AtTic20-IV *in roots are higher than those of *AtTic20-I*, while the opposite is true in leaf tissue. It can be speculated that the observed expression pattern reflects tissue-specific differentiation of both genes. *AtTic20-IV *may still partially complement for the function of *AtTic20-I*, as becomes evident from the viability of *attic20-I *knockout plants and the yellowish phenotype of *attic20-I *mutants overexpressing AtTic20-IV [26,27]. However, the severe phenotype of *attic20-I *plants, in conjunction with the observed differential expression pattern, clearly indicates specific functions of the two homologs. Furthermore, a higher *AtTic110 *expression rate as observed in antisense *attic20-I *lines might indicate another possible compensatory effect [19].

The expression pattern of the three investigated genes was found to be similar in *Arabidopsis *growing hydroponically with or without sucrose (Figure 1B) or on soil (data not shown). However, gene expression was generally higher in plants growing without sucrose.

### Tic20 protein is much less abundant than Tic110 in envelope membranes

**Figure 2:**
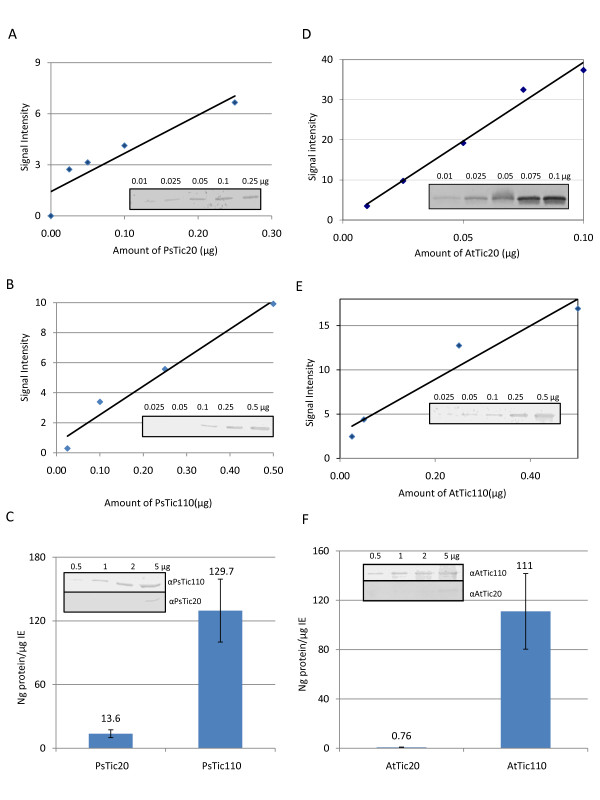
**Protein levels of Tic20 and Tic110 in envelope membranes**. Semi-quantitative analysis of Tic20 and Tic110 protein levels in (A-C) *Pisum sativum *(Ps) and (D-F) *Arabidopsis thaliana *(At). A dilution series of purified PsTic20, PsTic110, AtTic20 and AtTic110 was quantified after immunodetection with specific antibodies (A, B, D and E in inset). Calibration curves were calculated using known concentrations of proteins plotted against the quantified data (A, B, D and E). These curves were used to determine the amount of Tic20 and Tic110 in (C) pea and (F) *Arabidopsis *envelope samples. Insets in (C) and (F) show dilution series of corresponding envelopes after immunodetection with the indicated antibody. Presented data are the average of two independent experiments; a representative result is depicted.

Semi-quantitative analysis of Tic20 and Tic110 on protein level was performed using immunoblots of envelope membranes isolated from two-week-old pea and four-week-old *Arabidopsis *plants. In parallel, calibration curves were generated using a series of known concentrations of overexpressed and purified proteins (Figure 2A, B, D and 2E). After quantification of immunoblots from envelopes, amounts of PsTic20, PsTic110, AtTic20 and AtTic110 were determined using the corresponding calibration curve. The amount of PsTic110 in IE was found to be almost eight times higher than that of PsTic20 (Figure 2C), which differs strikingly from the similar transcript levels of the two genes detected in leaves (Figure 1A), indicating profound differences in posttranslational processes such as translation rate and protein turnover. In *Arabidopsis*, the absolute amount of AtTic110 is nearly the same as in pea (Figure 2F), however, *Arabidopsis *envelopes represent a mixture, containing both outer and IE vesicles. Thus, the relative amount of AtTic110 is possibly higher than in pea. Surprisingly, the amount of AtTic20 is more than 100 times lower than that of AtTic110, showing an even greater difference in comparison to the observed RNA expression levels (Figure 2F). Taking the different molecular size of Tic110 and Tic20 into account (~5:1), we still observe 20 times more AtTic110 than AtTic20 protein. In pea, we found 1.4 times more Tic110 RNA than Tic20, whereas in *Arabidopsis *the ratio of Tic110 to Tic20 is 20.3. The number of channel forming units must even be more different, since Tic110 was shown to form dimers [11], whereas Tic20 builds very large complexes between 700 kDa (this study) and 1 MDa [20]. Thus, two Tic110 molecules would be necessary to form a channel in contrast to Tic20, which would require many more molecules to form the pore. Though we cannot exclude that Tic20 might be subject to degradation by an unknown protease in vivo, protease treatments with thermolysin of right-side out IE vesicles in vitro clearly shows that Tic20 is very protease resistant, even in the presence of detergent. In contrast, Tic110 is easily degraded already without addition of detergent (Additional file 1). This argues against more rapid degradation of Tic20 compared to Tic110 during preparation of IE. The difference in Tic110 to Tic20 ratios both on the RNA and protein level between pea and *Arabidopsis *may be due to the different age of the plants or the different needs under the given growth conditions, and suggests that there is no strict stoichiometry between the two proteins. Moreover, the low abundance of Tic20 in comparison to Tic110 in envelopes (see also additional file 2) clearly demonstrates that Tic20 cannot be the main channel of the Tic translocon as previously suggested [21,24], since it cannot possibly support the required import rates of some highly abundant preproteins that are needed in the chloroplast.

### Tic20 forms high molecular weight complexes separately from Tic110

Experimental data suggested a common complex between Tic110 and Tic20 in chloroplast envelope membranes using a cross-linking approach [21]. However, the interaction was not visible in the absence of Toc components, making a stable association unlikely. Furthermore, no evidence for a common complex was found by Kikuchi *et al. *[20] using solubilized chloroplasts of pea and *Arabidopsis *for two-dimensional blue native/SDS-PAGE (2D BN/SDS-PAGE) analysis. Likewise, the difference in Tic110 to Tic20 ratios both on the RNA and protein level between pea and *Arabidopsis *indicates that a common complex, in which both proteins cooperate in translocation channel formation in a reasonable stoichiometry, is improbable.

**Figure 3:**
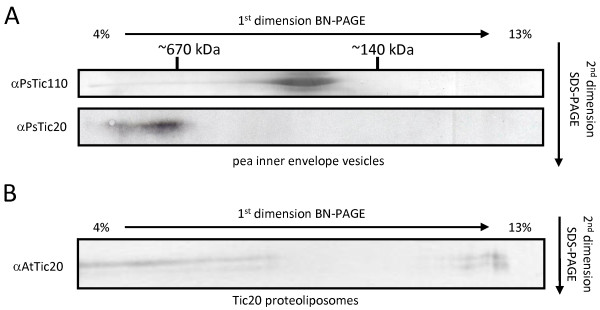
**Complex formation of Tic20 in inner envelope membranes and proteoliposomes**. Two-dimensional BN/SDS-PAGE of (A) inner envelope vesicles of *Pisum sativum *(Ps, 100 μg protein) and (B) AtTic20-proteoliposomes (20-30 μg protein). Samples were solubilized in 5% digitonin and separated by 4-13% BN-PAGE followed by 12.5% SDS-PAGE. Indicated specific antibodies were used for immunodetection. Representative results are depicted. At - *Arabidopsis thaliana*.

To clarify this issue, we addressed these partly conflicting results by using IE vesicles, which should minimize the possible influence of the interaction with Toc components on complex formation. Pea IE vesicles were solubilized in 5% digitonin and subjected to 2D BN/SDS-PAGE. Immunoblots revealed that both Tic20 and Tic110 are present in distinct high molecular weight complexes (Figure 3A): Tic110-containing complexes migrate at a size of ~ 200-300 kDa, whereas Tic20 displays a much slower mobility in BN-PAGE and is present in complexes exceeding 700 kDa, in line with the results from Kikuchi *et al. *[20]. However, at a similar molecular weight of 250 kDa on BN-PAGE not only Tic110 but also Hsp93, Tic62 and Tic55 were described [30]. The molecular weight of a complex containing all of these components would be much higher. Therefore, components of the Tic complex might associate with Tic110 very dynamically resulting in different compositions under different conditions, or alternatively, there are different complexes present at the same molecular weight.

An open question to date is the identity of possible interaction partners of Tic20 in the complex. Tic22, the only Tic component located in the intermembrane space, is a potential candidate, since both proteins were identified together in cross-linking experiments [21]. However, only minor amounts of Tic20 and Tic22 were shown to co-localize after gel filtration of solubilized envelope membranes [21]. A second candidate for common complex formation is PIC1/Tic21: Kikuchi *et al. *[20] demonstrated that a one-megadalton complex of Tic20 contains PIC1/Tic21 as a minor subunit. PIC1/Tic21 was proposed to form a protein translocation channel in the Tic complex, mainly based on protein import defects of knockout mutants and on structural similarities to amino acid transporters and sugar permeases [27]. An independent study by Duy *et al. *[31] favours the hypothesis that PIC1/Tic21 forms a metal permease in the IE of chloroplasts, rendering the import-related role questionable. This discrepancy will have to be addressed in the future.

To test the complex formation of Tic20 *in vitro *without the involvement of other proteins, we used Tic20-proteoliposomes for 2D BN/SDS-PAGE analysis, similarly to IE vesicles (Figure 3B). The migration behaviour of the protein resembles that observed in IE: the majority of the protein localizes in high molecular weight range, however, the signal appears more widespread and a portion is also detected at lower molecular weights, possibly as monomers. This observation reveals that Tic20 has the inherent ability to homo-oligomerize in the presence of a lipid bilayer. The less distinct signal could be due to different solubilization of Tic20 by digitonin in IE vesicles vs. liposomes, or could be an indication that additional subunits stabilize the endogenous Tic20 complexes, which are not present after the reconstitution. However, we interpret these observations as support for the hypothesis that the major component of the one megadalton complex in IE are homo-oligomers composed of Tic20.

### The N- and C-termini of Tic20 face the stromal side

**Figure 4:**
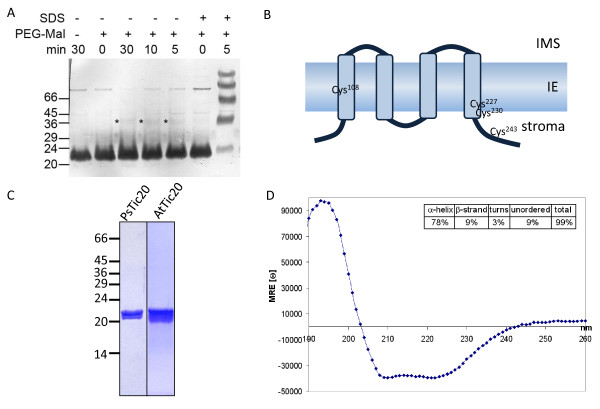
**Topology and secondary structure of Tic20**. (A) PEG-Mal labelling of *Pisum sativum *(Ps) inner envelope (IE) vesicles in the presence or absence of 1% SDS for the indicated times using a specific antibody against PsTic20 for immunodetection. Asterisks indicate a weak band most likely representing Tic20 with one labelled cystein (Cys) within the transmembrane region. A representative result of three repetitions is shown. (B) Topological model of Tic20 - indicating the position of Cys residues in PsTic20 - considering the PEGylation assay in (A) (based on structural prediction of TMHMM Server [23] and [25]). Boxes symbolise α-helical transmembrane domains (TM 1-4). IMS - intermembrane space. (C) The mature parts of Tic20 from *Pisum sativum *(PsTic20, amino acids 83-253) and *Arabidopsis thaliana *(AtTic20 amino acids 59-274) were overexpressed in an *E.coli *cell lysate system and in *E.coli *BL21 cells, respectively. Both proteins were purified by Ni^2+^-affinity chromatography. Coomassie-stained gels of representative purifications are shown. (D) Circular dichroism spectrum of overexpressed and purified PsTic20 in 20 mM Na-phosphate buffer (pH 8.0), 150 mM NaF, 0.8% Brij-35. The presented chromatogram is the average of three independent experiments. Secondary structure elements were quantified using the CDSSTR method from the DichroWeb server and results are presented in the inset.

*In silico *analysis of Tic20 predicts the presence of four hydrophobic transmembrane helices positioning both N- and C-termini to one side of the membrane (TMHMM Server [23] and [21,25]). According to these predictions, three cysteins (Cys) in PsTic20 face the same side, while the fourth would be located in the plane of the membrane. We used pea IE vesicles prepared in a right-side-out orientation [32] to determine the topology of Tic20 employing a Cys-labelling technique. To this end, the IE vesicles were incubated with a membrane-impermeable, Cys-reactive agent (metoxypolyethylenglycol-maleimide, PEG-Mal) that adds a molecular weight of 5,000 Da to the target protein for each reactive Cys residue. In our experiments PEG-Mal did not strongly label any Cys residues of Tic20 under the conditions applied (Figure 4A), indicating the absence of accessible Cys residues on the outside of the membrane. Only one faint additional band of higher molecular weight was detectable (Figure 4A, marked with asterisk), possibly due to a partially accessible Cys located within the membrane. In the presence of 1% SDS, however, all four Cys residues present in PsTic20 are rapidly PEGylated, as demonstrated by the appearance of four intense additional bands after only five minutes of incubation. The observed gain in molecular weight per modification is bigger than the expected 5 kDa for each Cys, but this can be attributed to an aberrant mobility of the modified protein in the Bis-Tris/SDS-PAGE used in the assay.

Our results support a four transmembrane helix topology in which both the C- and N-termini are facing the stromal side of the membrane (Figure 4B), with no Cys residues oriented towards the intermembrane space. Cys^108 ^is most likely located in helix one, Cys^227 ^and Cys^230 ^are oriented to the stromal side of helix four and Cys^243 ^is located in the stroma. This topology is also in line with green fluorescent protein-labelling studies by van Dooren *et al. *[22] indicating that the N- and C-termini also of the *Toxoplasma gondii *homolog of Tic20 face the stromal side of the inner apicoplast membrane.

### Tic20 is mainly α-helical

Tic20 was identified more than a decade ago but since then no heterologous expression and purification procedure has been reported, which could successfully synthesize folded full-length Tic20. Here, we report two efficient *Escherichia coli *(*E. coli*) based systems for Tic20 expression and purification from both pea and *Arabidopsis*: codon optimized PsTic20 (Additional file 3) was overexpressed in a S12 cell lysate in presence of detergents, and AtTic20 overexpression was successfully accomplished by adaptation of a special induction system [33]. Following these steps, both pea and *Arabidopsis *proteins could be purified to homogeneity by metal affinity purification (Figure 4C).

Using the purified protein, we performed structural characterization studies of Tic20 by subjecting it to circular dichroism (CD) spectroscopy (Figure 4D). The recorded spectra of PsTic20, displaying two minima at 210 and 222 nm and a large peak of positive ellipticity centered at 193 nm, are highly characteristic of α-helical proteins, and thus demonstrate that the protein exists in a folded state after purification in the presence of detergent. The secondary structure of Tic20 was estimated by fitting spectra to reference data sets (DichroWeb server [34,35]) resulting in an α-helical content of approximately 78%, confirming *in silico *predictions [21,25].

### Purified Tic20 protein inserts firmly into liposomes

**Figure 5:**
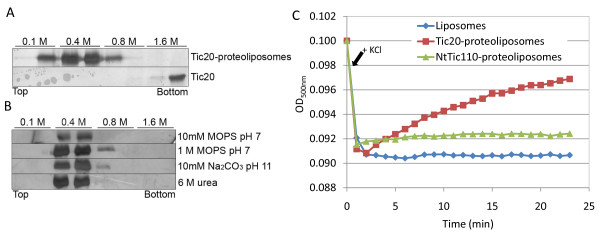
**Tic20 insertion into liposomes and channel formation**. (A) Flotation experiments of Tic20-proteoliposomes and Tic20 without vesicles in a sucrose gradient. Samples containing 1.6 M sucrose were loaded at the bottom of a sucrose step gradient and centrifuged to equilibrium (100,000 g, 19 h, 4°C). Fractions were analysed by silver-staining. (B) Flotation experiments of Tic20-proteoliposomes (similar to (A)) incubated under the indicated buffer conditions for 30 min at 4°C before centrifugation. (C) Swelling assay of liposomes, Tic20-proteoliposomes and NtTic110-proteoliposomes containing 20 mM Tris-HCl (pH 8.0), 100 mM NaCl. Change in optical density was measured at 500 nm (OD_500 nm_) of 1 ml solutions every minute. Arrow indicates the addition of 300 mM KCl. Presented results are the average of at least five repetitions; standard deviations were within 1.5-3%.

To better characterize Tic20 in a membrane-mimicking environment, heterologously expressed and purified AtTic20 was reconstituted into liposomes *in vitro*. Initially, flotation experiments were performed to verify a stable insertion. In the presence or absence of liposomes, Tic20 was placed at the bottom of a gradient ranging from 1.6 M (bottom) to 0.1 M (top) sucrose. In the presence of liposomes, Tic20 migrated to the middle of the gradient, indicating a change in its density caused by interaction with liposomes. In contrast, the protein alone remained at the bottom of the gradient (Figure 5A). Proteoliposomes were also treated with various buffers before flotation (for 30 min at 4°C), to test whether the protein is firmly inserted into the liposomal membrane or just loosely bound to the vesicle surface. None of the applied conditions (control: 10 mM MOPS/Tris, pH 7; high ionic strength: 1 M MOPS/Tris, pH 7; high pH: 10 mM Na_2_CO_3_, pH 11; denaturing: 6 M urea in 10 mM MOPS/Tris, pH 7) changed the migration behaviour of Tic20 in the gradient (Figure 5B), indicating that Tic20 was deeply inserted into the liposomal membrane. Thus, proteoliposomes represent a suitable *in vitro *system for the analysis of Tic20 channel activity.

### Tic20 forms a channel in liposomes

Even though Tic20 has long been suggested to form a channel in the IE membrane, this notion was solely based on structural analogy to other four-transmembrane helix proteins [21,24], and no experimental evidence has been provided so far. To investigate whether Tic20 can indeed form an ion channel, Tic20-proteoliposomes were subjected to swelling assays (Figure 5C). Changes in the size of liposomes in the presence of high salt concentrations, as revealed by changes in the optical density, can be used to detect the presence of a pore-forming protein [36]. After addition of 300 mM KCl to liposomes and Tic20-proteoliposomes, their optical densities dropped initially, due to shrinkage caused by the increased salt concentration [37]. However, the optical density of protein-free liposomes remained at this low level, showing no change in their size; whereas in the case of Tic20-proteoliposomes the optical density increased constantly with time. The increase in optical density (and therefore size) strongly supports the presence of a channel in Tic20-proteoliposomes that is permeable for ions, thereby creating an equilibrium between the inner compartment of the proteoliposomes and the surrounding buffer.

To exclude the possible effects of (i) contaminating channel-forming proteins derived from the bacterial membrane and (ii) a protein inserted into the liposomes (but not forming a channel), a further negative control was set up: Tic110 containing only the first three transmembrane helices (NtTic110) was purified similarly to Tic20 and reconstituted into liposomes. We chose this construct, since NtTic110 inserts into the membrane during *in vitro *protein import experiments [10]. Furthermore, as the full length and N-terminally truncated Tic110 possess very similar channel activities [11,12], it is unlikely that the N-terminal part alone forms a channel. The insertion of NtTic110 into liposomes was confirmed by incubation under different buffer conditions (high salt concentration, high pH and 6 M urea) followed by flotation experiments, similarly to Tic20 (data not shown). However, these NtTic110-proteoliposomes behaved similarly to the empty liposomes during swelling assays: after addition of salt, the optical density decreased, and except for a small initial increase, it remained at a constant level (Figure 5C). This makes it unlikely that a contamination from *E. coli *or simply the insertion of a protein into the liposomes caused the observed effect in the optical density of Tic20-proteoliposomes.

**Figure 6:**
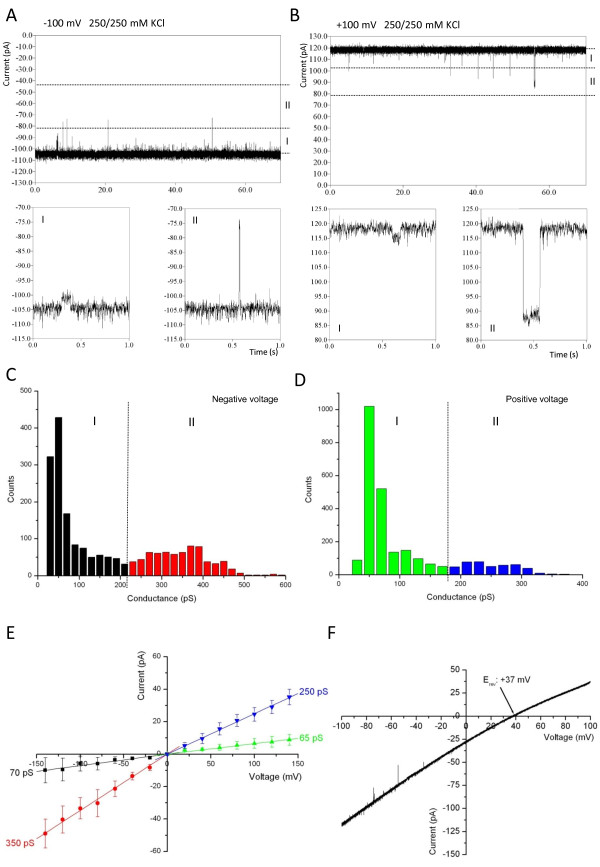
**Electrophysiological characterization of Tic20**. (A) and (B) Current traces of a Tic20 channel in lipid bilayer at -100 mV and +100 mV, respectively. Dotted lines indicate thresholds of each conductance class (I and II). Lower panels show representative gating events belonging to each class. (C) and (D) Conductance histograms of all gating events of Tic20 at negative and positive voltages, respectively. Colours represent different conductance classes (I and II). (E) Current-voltage relationship diagram of all analysed gating events ordered in the four indicated conductance classes using the same colour code as in (C) and (D). Indicated conductance values correspond to the slope of fitted linears in each class. (F) A representative voltage ramp of Tic20 demonstrating the cation selectivity of the channel with a positive reverse potential (E_rev_). Measurements were performed under symmetrical (A)-(E) and asymmetrical (F) buffer conditions (20 mM MOPS/Tris (pH 7.0), 250 mM and 20/250 mM KCl, respectively). Presented data derive from two independent fusions accounting for more than 4500 gating events and 16 voltage ramps.

To further characterize the channel activity of Tic20, electrophysiological measurements were performed. After the fusion of Tic20-proteoliposomes with a lipid bilayer, ion channel activity was observed (Figure 6A, B). The total conductance under symmetrical buffer conditions (10 mM MOPS/Tris (pH 7.0), 250 mM KCl) was dependent on the direction of the applied potential: 1260 pS (± 70 pS) and 1010 pS (± 50 pS) under negative and positive voltage values, respectively. The channel was mostly in the completely open state, however, individual single gating events were also frequently observed, varying in a broad range between 25 pS to 600 pS (Figure 6A-D). All detected gating events were depicted in two histograms (Figure 6C, D for negative and positive voltages, respectively). Two conductance classes (I and II) were defined both at negative and positive voltage values with thresholds of 220 pS and 180 pS, respectively (Figure 6A-E). Note that gating events belonging to the smaller conductance classes (I) occurred more frequently. The observed pore seems to be asymmetric, since higher conductance classes notably differ under positive and negative voltages. This is probably due to interactions of the permeating ions with the channel, which presumably exhibits an asymmetric potential profile along the pore. Since small and large opening events were simultaneously observed in all experiments, it is very unlikely that they belong to two different pores.

The selectivity of Tic20 was investigated under asymmetric salt conditions (10 mM MOPS/Tris (pH 7.0), 250/20 mM KCl). Similarly to the conductance values, the channel is intrinsically rectifying (behaving differently under negative and positive voltage values), supporting asymmetric channel properties. The obtained reverse potential is 37.0 ± 1.4 mV (Figure 6D). According to the Goldman-Hodgkin-Katz approach, this corresponds to a selectivity of 6.5:1 for K^+^:Cl^-^-ions, thus indicating cation selectivity similar to Tic110 [11].

To determine the channel's orientation within the bilayer, two side-specific characteristics were taken into account: the highest total conductance under symmetrical buffer conditions was measured under negative voltage values, and the channel rectifies in the same direction under asymmetrical buffer conditions (see voltage ramp, Figure 6D). Therefore, it seems that the protein is randomly inserted into the bilayer.

The pore size was roughly estimated according to Hille *et al. *[38]. Considering the highest conductance class (350 pS), a channel length of 1-5 nm and a resistivity of 247.5 Ω cm for a solution containing 250 mM KCl, taking into account that the conductivity of the electrolyte solution within the pore is ~5 times lower than in the bulk solution [39], the pore size was estimated to vary between 7.8-14.1 Å. This is in good agreement with the size of protein translocation channels such as Toc75 (14-26Å, [40]) in the outer envelope membrane and Tic110 (15-31 Å, [12]) in the IE. Thus, the size of the Tic20 pore would be sufficient for the translocation of precursor proteins through the membrane.

NtTic110, as a negative control, did not show any channel activity during electrophysiological measurements, indicating that the measured channel is not the result of a possible bacterial contamination (data not shown).

Considering our data presented here and those published in previous studies, we can conclude that the Tic translocon consists of distinct (at least two) translocation channels: On the one hand, Tic110 forms the main translocation pore and therefore facilitates import of most of the chloroplast-targeted preproteins; on the other hand, Tic20 might facilitate the translocation of a subset of proteins. This scenario would match the one found in the inner mitochondrial membrane, where specific translocases exist for defined groups of precursor proteins: the import pathway of mitochondrial carrier proteins being clearly separated from that of matrix targeted preproteins [41]. The situation in chloroplasts does not seem as clear-cut, but an analogous separation determined by the final destination and/or intrinsic properties of translocated proteins is feasible.

The severe phenotype of *attic20-I *mutants prompts us to hypothesize that Tic20 might be specifically required for the translocation of some essential proteins. According to cross-linking results [21], Tic20 is connected to Toc translocon components. Therefore, after entering the intermembrane space via the Toc complex, some preproteins might be transported through the IE via Tic20. On the contrary, Kikuchi *et al. *[20] presented that Tic20 migrates on BN-PAGE at the same molecular weight as the imported precursor of the small subunit of Rubisco (pSSU) and that *tic20-I *mutants display a reduced rate of the artificial precursor protein RbcS-nt:GFP. The authors interpreted these results in a way that Tic20 might function at an intermediate step between the Toc translocon and the channel of Tic110. However, being a substantial part of the general import pathway seems unlikely due to the very low abundance of Tic20. It is feasible to speculate that such abundant proteins as pSSU, which are imported at a very high rate, may interact incidentally with nearby proteins or indifferently use all available import channels. To clarify this question, substrate proteins and interaction partners of Tic20 should be a matter of further investigation.

Additionally, a very recent study [26] suggested AtTic20-IV as an import channel working side by side with AtTic20-I. However, detailed characterization of the protein (e.g. localization, topology) and experimental evidence for channel activity are still missing.

## Conclusions

**Figure 7:**
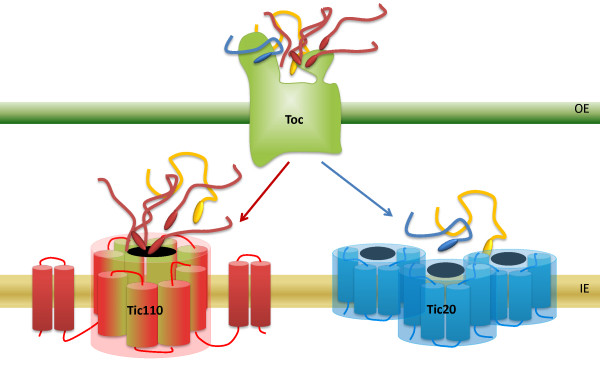
**Two independent channels at the Tic translocon**. Hypothetical model of Tic20 and Tic110 channels in the inner envelope (IE) of chloroplasts. After passing the Toc complex in the outer envelope (OE), preproteins are imported either via Tic110 (red) or Tic20 (blue) through the IE. Hypothetical precursors that might use both channel proteins are depicted in yellow. Tic110 is thought to form a homodimer with a total of eight amphipathic transmembrane helices forming the translocation channel and four hydrophobic α-helices involved in the insertion into the membrane (according to [8] and [11]). The proposed Tic20 channel is depicted as a homo-oligomer (only three molecules are shown, however, it is > 700 kDa) and, based on the low overall abundance, might be responsible for the import of a subset of preproteins, indicated by a smaller number of depicted precursors waiting at the entrance of the channel.

In this study we could clearly demonstrate that Tic20 and Tic110 function separately from each other, based on their different stoichiometry and their independent complex formation in IE vesicles. We furthermore present the first experimental evidence for Tic20 channel function. The very low abundance of Tic20 compared to Tic110 argues against Tic20 forming a major protein translocation channel, which would import the large number of preproteins that are needed in the chloroplast. Therefore, our data favour the idea that the Tic translocon comprises at least two translocation channels: Tic110, constituting the main import channel [11], and Tic20, which might import a special subset of preproteins (a hypothetical model of the two Tic translocation channels is depicted in Figure 7). A similar system exists in the inner membrane of mitochondria, where the TIM22 and TIM23 complexes mediate the import of different sets of proteins [41]. Unfortunately, due to the lethality of *tic110 *and the very severe phenotype of *tic20-I *homozygous knockout mutants, their separate mode of action will be very difficult to investigate *in vivo*.

## Methods

### Plant growth conditions

Pea plants (*Pisum sativum *var. Arvica) were grown under a 14-h light/10-h dark regime at 20°C/15°C. *Arabidopsis thaliana *plants (ecotype Columbia) were grown either on soil under the conditions described in Benz *et al. *[42] or hydroponically. The latter were cultured under non-sterile conditions as modified from [43]. Briefly, after expansion of their first true leaves, seedlings were transferred from plates (containing 0.5 × MS with 1% sucrose) into a GA-7 Magenta vessel (Sigma-Aldrich) onto a one cm thick cut sponge. The nutrient solution contained 1 mM KH_2_PO_4_, 0.5 mM MgSO_4_, 0.25 mM CaSO_4_, 20 μM Fe-EDTA, 25 μM H_3_BO_3_, 2 μM ZnSO_4_, 2 μM MnSO_4_, 0.5 μM CuSO_4_, 0.5 μM (NH_4_)_6_Mo_7_O_24 _and 0.5 mM NH_4_NO_3 _in the presence or absence of 1% sucrose. The growth chambers were kept under 16-h light/8-h dark regime at 21°C/16°C for four weeks. Plants were either harvested from the dark or before noon from growth light. Material was usually used immediately and fresh. If not possible, leaf material was shock-frozen in liquid nitrogen and stored at -80°C until use.

### qRT-PCR

RNA isolation, cDNA preparation, qRT-PCR and data analysis were performed essentially as described in [44]. Gene-specific primers were constructed for *PsTic20 *[GenBank: AF095285.1], *PsTic110 *[GenBank: Z68506.1], *AtTic20-I *[TAIR: At1g04940.1], *AtTic20-IV *[TAIR: At4g03320.1] and *AtTic110 *[TAIR: At1g06950.1] (Table 1). All reactions were performed in quadruplicates.

**Table 1:** Primers for qRT-PCR analysis

primer	forward	reverse
PsTic20	CCTAGATGGTCTCTCATAGC	GCAGTAGTCCAGAAATGC

PsTic110	CAAGGAAACTGCTCTGTC	CTCCTTTGATGTCCTCTACC

Ps18SrRNA	CCAGGTCCAGACATAGTAAG	GAGGGTTACCTCCACATAG

AtTic20-I	AGGTTATAGGGACCGTTAGC	CTTAGTCGTACGGAATCTGG

AtTic20-IV	CTATGTCCAACCTTTTCTCG	CTGTTTCAAGAAGCATACCC

AtTic110	CTAAAGGAGTGGTCTTGTCG	GCAGAAGATAATGCTCCATC

At18SrRNA	AACTCGACGGATCGCATGG	ACTACCTCCCCGTGTCAGG

Gene-specific primers generated for *PsTic20*, *PsTic110*, *Ps18SrRNA*, *AtTic20-I*, *AtTic20-IV*, *AtTic110 *and *At18SrRNA *applied in the qRT-PCR analysis.

### Isolation of envelope vesicles

Membrane fractions enriched in right-side-out IE vesicles of pea chloroplast membranes were isolated from intact chloroplasts of 10 to 12-day-old pea plants as described previously [32]. For *Arabidopsis *envelope preparation, chloroplasts were first isolated from 4-week-old soil-grown plants from the dark as described by Seigneurin-Berny *et al. *[45]. Chloroplasts were subsequently resuspended in 15 ml of 10 mM HEPES-KOH, pH 7.6, 5 mM MgCl_2_, and lysed using 50 strokes in a small (15 ml) Dounce tissue grinder (Wheaton Science Products, Millville, NJ, USA). Further separation into stroma, thylakoids and envelopes was performed according to Li *et al. *[46].

### Protein expression and purification

The sequence coding for the mature part of Tic20 from *Pisum sativum *(PsTic20, amino acids 83-253) was codon optimized with the Leto 1.0 program by Entelechon (Regensburg, Germany) (Additional file 3). The optimized gene was then synthesized by Entelechon and finally cloned into pIVEX2.3 (Roche, Germany). The mature part of *Arabidopsis thaliana *Tic20-I (AtTic20, amino acids 59-274) was cloned into pCOLDII (Takara-Bio, Kyoto, Japan). The mature part of Tic110 from *Pisum sativum *without the N-terminal hydrophobic domain (PsTic110, amino acids 122-996) and a similar construct for the homologous part from *Arabidopsis thaliana *Tic110 (AtTic110, amino acids 141-1016) were cloned into pET21d [11]. The same expression vector was used for the cloning of the N-terminal part of mature Tic110 (NtTic110, amino acids 76-258) from *Arabidopsis thaliana*.

The codon optimized PsTic20 was overexpressed in a self-made *E.coli *cell-free lysate system (S12) which was prepared essentially as described by Kim *et al. *[47]. Shortly, expression of soluble PsTic20 was carried out at 30°C for 1-2 h with constant rolling in 100-200 μl reaction mixture (57 mM Hepes-KOH (pH 8.2), 1.2 mM ATP, 0.65 mM cAMP, 0.85 mM each of CTP, GTP and UTP, 2 mM DTT, 90 mM potassium glutamate, 80 mM ammonium acetate, 15 mM magnesium acetate, 34 μg/ml _L_-5-formyl-5,6,7,8-tetrahydrofolic acid, 0.75 mM each of 20 amino acids, 2% polyethylene glycol 8000, 100 mM creatine phosphate, 0.27 mg/ml creatine kinase, 0.17 mg/ml *E. coli *total tRNA mixture (from strain MRE600), 10 μg/ml plasmid DNA, 25% BL21 (DE3) and 2% BL21 (DE3) RIL-pAR1219 cell extract and 0.8% Brij-35). After removing insoluble material (10,000 g, 4°C, 10 min) the supernatant was diluted 1:3 with 50 mM NaH_2_PO_4_-NaOH (pH 8.0), 300 mM NaCl, 0.8% Brij-35, 20 mM imidazole and purified using Ni-NTA-Sepharose (GE Healthcare, Munich, Germany).

For expression and purification of AtTic20/pCOLDII, transformed BL21 (DE3) cells (Novagen/Merck) were grown at 37°C in M9ZB medium to an OD_600 _of 0.4 and then shifted to 15°C for 30 min. After induction with 1 mM isopropyl-1-thio-β-D-galactopyranoside, cells were further grown at 15°C overnight. The harvested cells were resuspended in 50 mM Tris-HCl (pH 8.0), 150 mM NaCl, 5 mM dithiothreitol (DTT), lysed (M-110L Microfluidizer Processor, Microfluidics, Newton, MA, USA), pelleted (20,000 g, 4°C, 20 min) and solubilized in the presence of 1% *n*-lauroylsarcosine (N-LS) for 1 h at 4°C. Purification was carried out in the presence of 0.3% N-LS using Ni-NTA-Sepharose (GE Healthcare, Munich, Germany). PsTic110/pET21d was overexpressed and purified as described previously [11]. AtTic110/pET21d overexpression and purification was performed similarly to PsTic110. NtTic110 was overexpressed similarly to PsTic110, whereas its purification was performed similarly to AtTic20 except that in all buffers 300 mM NaCl was present.

### Immunoblotting

Immunoblotting was performed using polyclonal antisera from rabbits raised against heterologously overexpressed proteins, followed by incubation with monoclonal rabbit secondary antibody, visualized by alkaline phosphatase or by a chemiluminescence detection system (Pierce, Rockford, IL, USA). The antisera against atTic110 and psTic20 were purified against a poly-histidine matrix. To this end, Poly-L-Histidine was coupled to CNBr-activated sepharose (GE Healthcare) according to the manufacturer's recommendations. Antisera were diluted 3 times and incubated over night at 4°C with the Poly-His matrix. Sepharose beads were sedimented and the supernatant was used as purified serum in the immunoblots (see additional file 4).

### Semi-quantitative protein analysis

For semi-quantitative protein analysis, a dilution series of purified PsTic20, PsTic110, AtTic20 and AtTic110 was loaded on SDS-PAGE in parallel to a dilution series of pea IE vesicles and *Arabidopsis *mixed envelope membranes. After immunodetection with specific antibodies, the intensity of the resulting bands was quantified (AIDA Software). The band intensity of the purified proteins was first plotted against the known protein amount. This calibration curve was then applied to determine the amount of Tic20 and Tic110 present in the membrane samples. The analysis was repeated two times with different envelope preparations.

### Two dimensional BN/SDS-PAGE

BN-PAGE was performed essentially as described by Schaegger and von Jagow [48] and Küchler *et al. *[30] with minor modifications. IE membranes (50-200 μg protein) or Tic20-proteoliposomes (30 μg protein) were solubilized in 50 mM Bis-Tris/HCl (pH 7.0), 750 mM 6-aminocaproic acid and 5% digitonin. After incubation on ice (IE) or at room temperature (liposomes) for 15 min, samples were centrifuged at 256,000 g for 10 min at 4°C. The supernatant was supplemented with 0.1 volume of a Coomassie Blue G solution (5% Coomassie Brilliant Blue G-250, 750 mM 6-aminocaproic acid) and loaded on a polyacrylamide gradient gel. Following the first dimension, lanes were incubated sequentially in 1% SDS, 1 mM β-mercaptoethanol (β-ME), in 1% SDS without β-ME and in SDS-PAGE running buffer (25 mM Tris, 192 mM glycine, and 0.1% SDS) at room temperature for 15 min each and then horizontally subjected to a second dimension SDS-PAGE. After separation, immunodetection was performed.

### CD-spectroscopy

Purified PsTic20 was dialysed against 20 mM Na_2_HPO_4_/NaH_2_PO_4 _buffer (pH 8.0), 150 mM NaF, 0.8% Brij-35 prior to CD analysis. Experiments were carried out at 20°C using a J-810 spectropolarimeter (Jasco, Groβ-Umstadt, Germany) flushed with N_2_. Spectra were collected from 260 to 190 nm using a 1 mm path length of a cylindrical quartz cell. Each spectrum was the average of three scans taken at a scan rate of 20 nm/min with a spectral bandwidth of 1 nm. The experiment was repeated three times in a concentration range of 0.02 to 0.284 mg/ml protein. For the final representation, the baseline was subtracted from the spectrum. The analysis was performed using the CDSSTR method from the DichroWeb server [34,35].

### PEGylation assay

IE vesicles were treated with 10 mM metoxypolyethylenglycol-maleimide 5000 Da (PEG-Mal, Laysan Bio, Arab, AL, USA) in a buffer containing 100 mM Tris-HCl (pH 7.0), 1 mM EDTA, for the indicated times at room temperature in the dark in absence or presence of 1% SDS. The PEGylation reaction was stopped by addition of 100 mM DTT and SDS-PAGE sample buffer. NuPAGE Bis-Tris gels (10% acrylamide) were employed using a MES running buffer. Tic20 was detected by immunoblotting.

### Liposome preparation and flotation assay

Proteoliposomes of AtTic20 and NtTic110 were prepared as described previously [11]. To prepare unilamellar liposome vesicles, samples were extruded 21 times through a 200 nm polycarbonate filter (Liposofast, Avestin, Ottawa, Canada). Purified AtTic20 (in 20 mM Tris-HCl pH 8.0, 150 mM NaCl, 0.3% N-LS) (or NtTic110 or buffer as controls) was mixed with liposomes and incubated for 1.5 h at 4°C. The samples were dialysed for 16 h at 4°C against a buffer without detergent (20 mM Tris-HCl pH 8.0, 100 mM NaCl) and the remaining detergent was removed during 2 h incubation at 4°C with Bio-Beads SM-2 (Bio-Rad Laboratories, Hercules, USA). Liposome-associated and liposome-free proteins were separated by flotation through a sucrose gradient, similar to Balsera *et al. *[11]: Samples were adjusted to a sucrose concentration of 1.6 M (1 ml, bottom) and overlaid with 3 ml of step sucrose gradient (0.8, 0.4 and 0.1 M, top). After centrifugation (100,000 g, 19 h, 4°C) 0.5 ml fractions were collected and precipitated with trichloroacetic acid (TCA). The samples were resuspended in Laemmli-buffer, separated by SDS-PAGE and detected by silver-staining.

### Swelling assay

Freshly prepared liposomes and proteoliposomes (AtTic20 and NtTic110) were diluted to 1 ml to a starting optical density of approximately 0.1 at 500 nm. The optical density of the samples was measured with a Shimadzu UV-2401PC Spectrophotometer (Columbia, USA) for the indicated time. At the beginning of the measurements 300 mM KCl was added to the samples. Experiments were repeated at least five times.

### Electrophysiological measurements

Electrophysiological measurements were performed using the IonoVation Bilayer Explorer (Osnabrück, Germany) according to suppliers instructions. Proteoliposomes were fused with the bilayer by applying an osmotic gradient of 250/20 mM KCl between the two chambers separated by the bilayer (the sample was added to the *cis *chamber). Conductance was measured under symmetric buffer conditions (10 mM MOPS/Tris (pH 7.0), 250 mM KCl) at 15 different voltage values in a step gradient from -140 mV to +140 mV applying each voltage value for 5 min. Selectivity was tested under asymmetric buffer conditions (10 mM MOPS/Tris (pH 7.0), 250/20 mM KCl) with voltage values changing in a linear gradient from -100 mV to +100 mV and *vice versa*, eight times for each fusion.

For analysis, AxoScope 10.2 (Axon Instruments, Union City, USA), Ephys 5.0 (made by Thomas Steinkamp, University of Osnabrück) in combination with Origin 7.0 (OriginLab Corporation, Northampton, MA, USA) and Microsoft Excel 2007 softwares were used. Presented data are derived from two independent fusion events.

## Authors' contributions

JPB developed the expression and purification procedures for Tic20, carried out the qRT-PCR and semi-quantitative immunoblot analyses, performed the two-dimensional BN/SDS-PAGE, as well as the topological characterization of Tic20 by PEGylation and CD-spectroscopy. EKB carried out all proteoliposome assays including the electrophysiology of Tic20 and drafted the manuscript. JS conceived of the study and participated in its design and coordination. BB participated in the design and coordination of the study. All authors read and approved the final manuscript.

## Supplementary Material

Additional file 1 **Thermolysin treatment of inner envelope vesicles**. Right side-out IE vesicles were treated for the indicated times with the protease thermolysin (1 μg/10 μg inner envelope). 1% Tx100 indicates the presence of 1% Triton X100 during the treatment. Proteolysis was terminated by EDTA and the samples analyzed by immunodetection with antibodies against Tic110, Tic62 and Tic20. Click here for file

Additional file 2 **Coomassie-stained samples of inner and outer envelope vesicles**. 20 μg of *Pisum sativum *outer and inner envelope vesicles, respectively, were loaded onto a 12.5% SDS-PAGE gel and stained with Coomassie Blue. Tic110 and Toc75 are indicated by asterisks. The region where Tic20 should be located is marked by a bracket. Click here for file

Additional file 3 **Sequence alignment of PsTic20 with its codon-optimized form**. Sequence alignment was performed with the cDNA sequence of the mature *Tic20 *from *Pisum sativum *(*PsmTic20*, amino acids 83-253) and the codon-optimized form (*PsmTic20-opt*) obtained from Entelechon (Regensburg, Germany). Identical nucleotides are shaded by black boxes. Click here for file

Additional file 4 **Test of antibodies against the His-tag**. (A) Indicated amounts of purified His-FNRL1 and 10 μg of *Pisum sativum *inner envelope vesicles were loaded onto SDS-PAGEs and blotted on nitrocellulose. Immunodetection with the indicated antisera revealed unspecific detection of the His-moiety by anti-PsTic20, anti-PsTic110 and anti-AtTic110. (B) anti-PsTic20 and anti-AtTic110 were purified against CNBr-coupled Poly-His and again tested for reactivity. (C) Indicated amounts of purified N-terminally His-tagged proteins A and B as well as 10 μg and 15 μg of AtEnv were loaded onto SDS-PAGEs and blotted on nitrocellulose. Immunodetection was performed with antiserum against AtTic20. The endogenous AtTic20 protein is indicated by an arrow. Click here for file
