# Supplementary figures and images for: Tic20 forms a channel independent of Tic110 in chloroplasts
Source: BMC Plant Biol. 2011 Sep 30;11:133. doi: 10.1186/1471-2229-11-133 (PMC3203047; doi:10.1186/1471-2229-11-133)

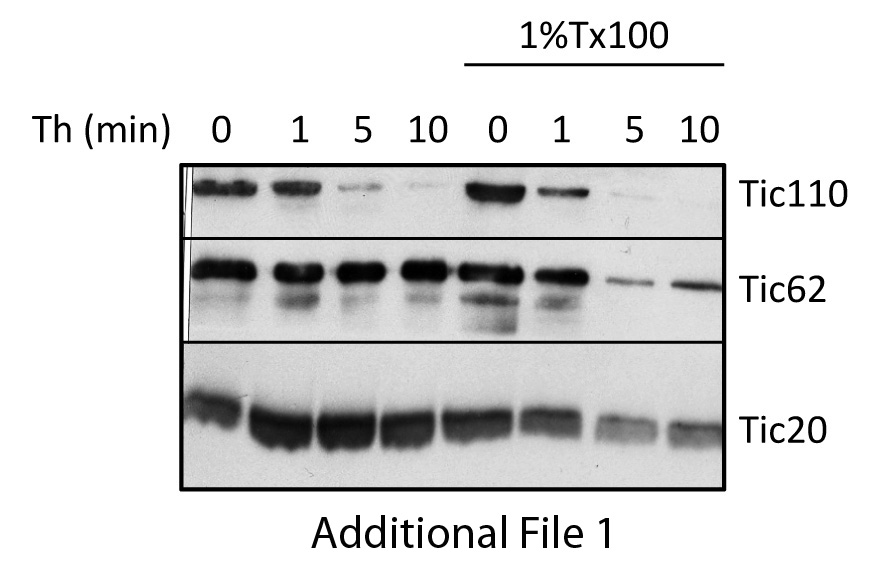

Supplement: Additional file 1 — Thermolysin treatment of inner envelope vesicles. Right side-out IE vesicles were treated for the indicated times with the protease thermolysin (1 μg/10 μg inner envelope). 1% Tx100 indicates the presence of 1% Triton X100 during the treatment. Proteolysis was terminated by EDTA and the samples analyzed by immunodetection with antibodies against Tic110, Tic62 and Tic20. [file 1471-2229-11-133-S1.JPEG]

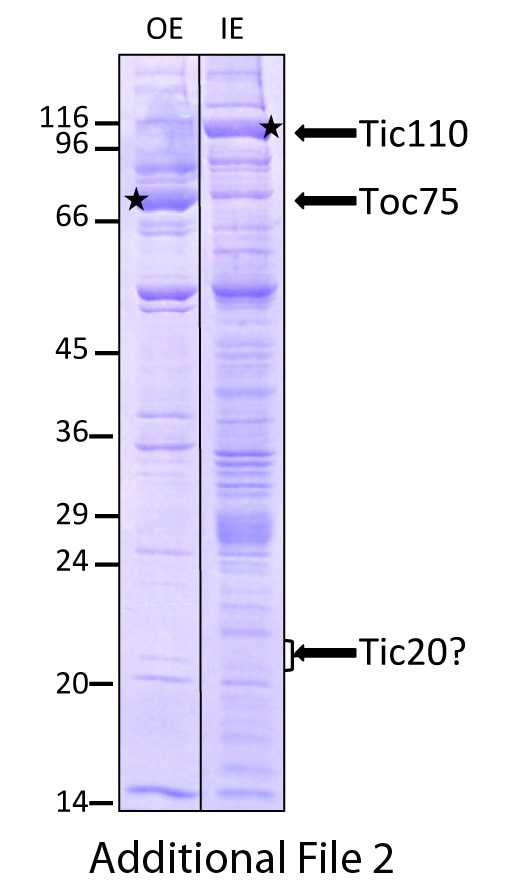

Supplement: Additional file 2 — Coomassie-stained samples of inner and outer envelope vesicles. 20 μg of Pisum sativum outer and inner envelope vesicles, respectively, were loaded onto a 12.5% SDS-PAGE gel and stained with Coomassie Blue. Tic110 and Toc75 are indicated by asterisks. The region where Tic20 should be located is marked by a bracket. [file 1471-2229-11-133-S2.JPEG]

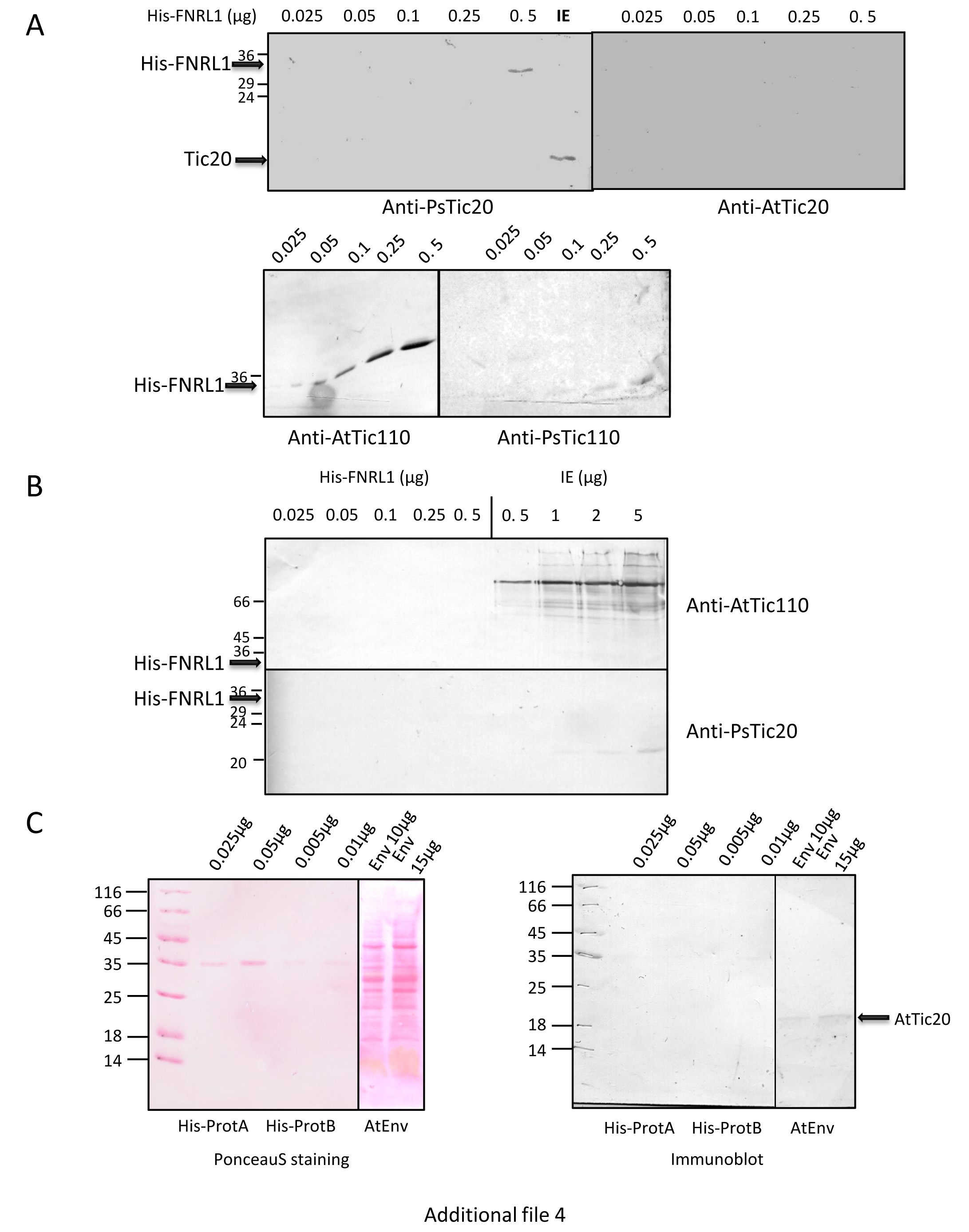

Supplement: Additional file 4 — Test of antibodies against the His-tag. (A) Indicated amounts of purified His-FNRL1 and 10 μg of Pisum sativum inner envelope vesicles were loaded onto SDS-PAGEs and blotted on nitrocellulose. Immunodetection with the indicated antisera revealed unspecific detection of the His-moiety by anti-PsTic20, anti-PsTic110 and anti-AtTic110. (B) anti-PsTic20 and anti-AtTic110 were purified against CNBr-coupled Poly-His and again tested for reactivity. (C) Indicated amounts of purified N-terminally His-tagged proteins A and B as well as 10 μg and 15 μg of AtEnv were loaded onto SDS-PAGEs and blotted on nitrocellulose. Immunodetection was performed with antiserum against AtTic20. The endogenous AtTic20 protein is indicated by an arrow. [file 1471-2229-11-133-S4.JPEG]
