# Supplementary material for: Tic20 forms a channel independent of Tic110 in chloroplasts
Source: BMC Plant Biol. 2011 Sep 30;11:133. doi: 10.1186/1471-2229-11-133 (PMC3203047; doi:10.1186/1471-2229-11-133)
Supplement: Additional file 3 — Sequence alignment of PsTic20 with its codon-optimized form. Sequence alignment was performed with the cDNA sequence of the mature Tic20 from Pisum sativum (PsmTic20, amino acids 83-253) and the codon-optimized form (PsmTic20-opt) obtained from Entelechon (Regensburg, Germany). Identical nucleotides are shaded by black boxes. [file 1471-2229-11-133-S3.DOC]

**Additional files**

**Additional file 3 - Sequence alignment of PsTic20 with its codon-optimized form**


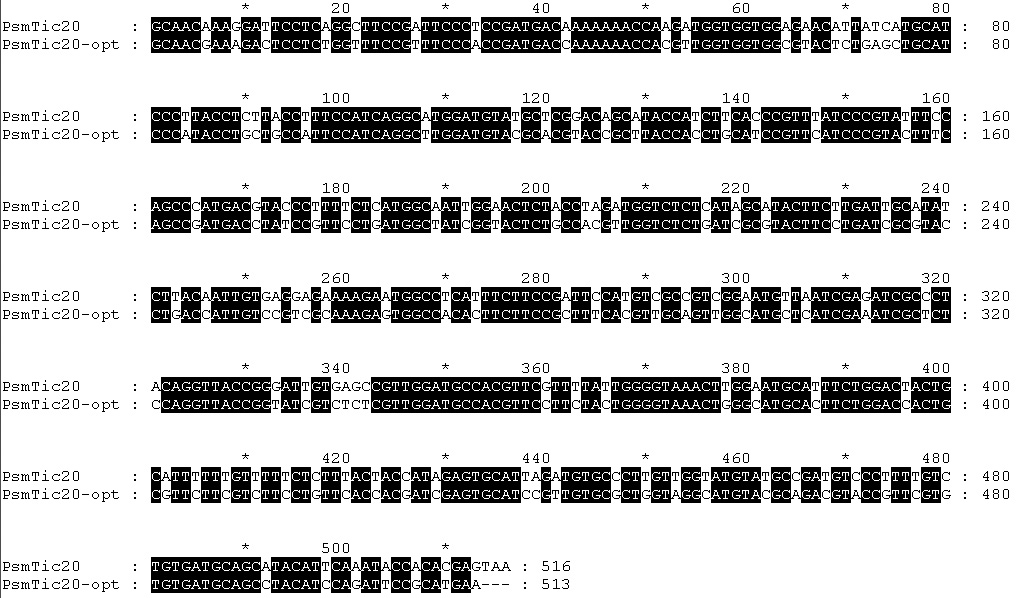
Sequence alignment was performed with the cDNA sequence of the mature *Tic20* from *Pisum sativum* (*PsmTic20*, amino acids 83-253) and the codon-optimized form (*PsmTic20-opt*) obtained from Entelechon (Regensburg, Germany). Identical nucleotides are shaded by black boxes.
